# Tissue Factor Expression in Penile Squamous Cell Carcinoma: A Potential Marker of HPV-Independent Disease

**DOI:** 10.3390/cancers17213410

**Published:** 2025-10-23

**Authors:** Jamaal C. Jackson, Andrew C. Johns, Leticia Campos Clemente, Christopher M. Manuel, Wei Qiao, Wei Lu, Khaja Khan, Luisa M. Solis Soto, Jad Chahoud, Priya Rao, Matthew T. Campbell, Curtis A. Pettaway, Niki M. Zacharias

**Affiliations:** 1 Department of Urology, University of Texas MD Anderson Cancer Center, Houston, TX 77030, USAcpettawa@mdanderson.org (C.A.P.); 2 Department of Genitourinary Medical Oncology, University of Texas MD Anderson Cancer Center, Houston, TX 77030, USA; acjohns@mdanderson.org (A.C.J.); mcampbell3@mdanderson.org (M.T.C.); 3 Department of Translational Molecular Pathology, University of Texas MD Anderson Cancer Center, Houston, TX 77030, USA; lcampos@mdanderson.org (L.C.C.); lmsolis@mdanderson.org (L.M.S.S.); 4 Department of Biostatistics, University of Texas MD Anderson Cancer Center, Houston, TX 77030, USA; cmmanuel@mdanderson.org (C.M.M.); wqiao@mdanderson.org (W.Q.); 5 Department of Genitourinary Oncology, Moffitt Cancer Center, Tampa, FL 33612, USA; jad.chahoud@moffitt.org; 6 Department of Pathology, University of Texas MD Anderson Cancer Center, Houston, TX 77030, USA; prao@mdanderson.org

**Keywords:** penile squamous cell carcinoma, tissue factor, HPV, p16, TROP2, nectin-4, aberrant p53

## Abstract

**Background/Objectives**: In a series of 33 patients with advanced penile squamous cell carcinoma (PSCC), we evaluated tissue factor (TF), TROP2, and nectin-4 protein expression as potential therapeutic targets. Expression levels of these proteins were also correlated to clinicopathological characteristics, including high-risk human papillomavirus (HPV), CDKN2A (p16) status, and aberrant p53 expression. **Methods**: A tissue microarray (TMA) was constructed with three cores per patient tumor (99 total cores). Anti-TF antibody staining was performed by immunohistochemistry, and H-scores for membrane and cytoplasm staining were assessed (range 0–300). The percentage of cores and patient tumors staining positive for TF (≥10% of tumor cells with at least 1+ intensity in cytoplasm and/or membrane) and H-scores were described and compared with HPV and p16 status. The association of TF expression with tumor grade, presence of metastatic disease, lymphovascular invasion (LVI), perineural invasion (PNI), aberrant p53 expression, recurrence-free survival (RFS), and cancer-specific survival (CSS) was assessed. Nectin-4 and TROP2 staining and their association with clinical/pathological data were determined in a similar manner. **Results**: TF staining was evident in 26 (81.3%) of the cohort and was more prominent in HPV-negative tumors in both the membrane (H-score 69.6 vs. 18.8; *p* = 0.003) and cytoplasm (H-score 59.2 vs. 17.7, *p* = 0.007). Cytoplasmic (H-score 61.7 vs. 11.7, *p* < 0.001) and membrane TF staining (H-score 71.7 vs. 15.0, *p* < 0.001) favored p16-negative tumors. The p53 status was more likely to be aberrant in the higher TF staining samples (cytoplasm H-score 61.7 vs. 18.3, *p* = 0.012; membrane H-score 67.5 vs. 20.3, *p* = 0.006). We observed an association with TROP2 staining and positive p16 status (membrane H-score 120.3 vs. 85, *p* = 0.052; cytoplasmic H-score 135 vs. 107.5, *p* = 0.041). We observed an association of TROP2 staining with positive LVI (membrane H-score 136.7 vs. 66.7, *p* = 0.014; cytoplasmic H-score 110 vs. 93.3, *p* = 0.04). We found no association between TF, TROP2, or nectin-4 staining with CSS or RFS; however, we suspect that this was due to our small sample size. **Conclusions**: Our results indicate that TF was expressed in the majority of advanced PSCC with enhanced expression among HPV-independent, p53-aberrant tumors and may represent a novel therapy target in advanced PSCC.

## Simple Summary

The aim of our study was to evaluate tissue factor (TF) expression in advanced penile squamous cell carcinoma (PSCC) and its correlation with clinicopathological characteristics and survival outcomes and as a potential target for therapeutic agents. We also explored the expression of nectin-4 and trophoblast cell-surface antigen (TROP2) in the same context. A tissue microarray (TMA) was constructed from a series of 33 patients’ primary PSCC lesions. The expression of TF, nectin-4, TROP2, and aberrant p53 was determined in the TMA. TF was expressed in 81.3% of specimens, and we found an increase in TF expression in HPV-independent, p16-negative, and p53-aberrant tumors, while TROP2 expression favored HPV-associated samples. We observed no association between expression of TF, TROP2, nectin-4, and patient survival (recurrence-free survival, cancer-specific survival). Our study suggests TF is a possible therapeutic target in advanced PSCC, especially among the HPV-independent p53 aberrant cohort.

## 1. Introduction

Penile squamous cell carcinoma (PSCC) is a rare yet aggressive malignancy with potentially devastating consequences. Patients with advanced PSCC who remain untreated typically die within 2 years due to complications from progressive locoregional or distant metastatic disease [1,2]. Treatment for locoregionally advanced disease consists of either neoadjuvant cisplatin-based chemotherapy (most often in the form of paclitaxel, ifosfamide, and cisplatin (TIP)) combined with consolidative surgery. Alternatively, among patients who are not candidates for combination chemotherapy regimens, radiation-based strategies, often using chemosensitization, can also be used [3,4,5]. In patients eligible for systemic therapy, roughly 57% of patients will have evidence of disease response, which yields a four-fold improvement in median overall survival compared with non-responders when combined with surgery [6]. There are limited treatment options for patients whose cancer progresses after TIP chemotherapy, and the estimated median overall survival of non-responders ranges from less than 6 months to 17 months depending on the disease burden and whether survival was assessed from the start of treatment or at the time of clinical progression [7,8,9]. In either case, the poor outcome of the chemo-refractory cohort highlights the dire need for novel therapies in this disease space.

There has been an increasing interest in exploring the use of antibody-drug conjugates (ADCs) as therapeutic options in advanced PSCC. Recent analyses in PSCC have found a number of potential cellular targets, such as nectin-4 and trophoblast cell-surface antigen 2 (TROP2), that are associated with increased expression in high-risk human papillomavirus (HPV)-associated PSCC when compared with HPV-negative cases [10,11]. High expression of these molecules may be linked to contrasting clinical implications. In a recent large cohort analysis, high nectin-4 expression correlated with improved cancer-specific survival (CSS), while increased TROP2 expression was associated with early disease progression [10].

Tissue factor (TF), also known as thromboplastin or coagulation factor III, is a perivascular transmembrane glycoprotein with many functions, including disruption of apoptosis via the Janus kinase (JAK)/signal transducer and activator of transcription (STAT) pathway [12]. Overexpression has been observed in numerous solid malignancies, including cervical, prostate, and bladder cancer, and is thought to spur primary tumor growth, neoangiogenesis, and tumor invasion [12,13]. The prevalence and importance to carcinogenesis make TF an intriguing potential therapeutic target. Multiple studies examining therapeutic targeting of TF in cervical cancer have shown improved efficacy and safety in the salvage setting when compared with standard therapy [14,15]. Furthermore, the commercial availability and FDA approval of a TF-targeting ADC make it worthwhile to evaluate this target in PSCC. In this study, we evaluated TF expression in advanced PSCC as a potential suitable therapy target and its correlation with clinicopathological characteristics and survival outcomes.

## 2. Materials and Methods

### 2.1. Patient Tissues

The current study was approved by the Institutional Review Board at The University of Texas MD Anderson Cancer Center (Protocol number 2022-0733). Patient consent was waived because the study involved no diagnostic or therapeutic intervention as well as no direct patient contact. Patients were selected from a 143-patient cohort previously described by Chahoud et al. [16]. These patients were further filtered to reflect those with more advanced disease by first selecting those with clinical or pathologic evidence of nodal metastasis before eliminating patients with ≤pathologic stage 1 disease. The cohort was ultimately selected to reflect an even distribution of HPV-associated and HPV-independent disease. Clinicopathologic data was collected, including baseline characteristics, patient outcomes, and follow-up information. HPV-positive status was determined by either HPV-ISH hybridization assay or by Cobas HPV testing as described by Chahoud et al. [16].

### 2.2. Pathological Analysis

The pathology reports in the electronic medical record were reviewed to identify appropriate archived tissue samples obtained during the primary tumor resection. Formalin-fixed paraffin-embedded (FFPE) “donor” blocks of the selected samples were obtained. The donor blocks were then sectioned to create microscopic slides stained with hematoxylin and eosin (H&E). The H&E-stained slides were reviewed by an MDACC genitourinary pathologist (P.R.), and areas of malignant cells were marked. Once samples with sufficient tumor representative of each patient were identified, the donor blocks were used to construct a tissue microarray (TMA) with three 1.0 mm cores per tumor. A microtome was utilized to cut 5 μm sections from the cores to create TMA slides for molecular analysis [17].

### 2.3. Immunohistochemistry (IHC) Staining

TROP2, nectin-4, p16, p53, and human tissue factor-1 (CD142) markers were studied with IHC assays. Four-micron-thickness formalin-fixed paraffin-embedded (FFPE) tissue sections were mounted on positively charged glass slides. The section went through standard staining protocols on either Leica BOND™ Autostainer (Leica Biosystems, Deer Park, IL, USA) with BOND™ Polymer Refine Detection kit (Leica Biosciences, DS9800) or Ventana Discovery Ultra (Ventana Medical System, Oro Valley, AZ, USA) with OptiView DAB Detection Kit (Roche, Basel, Switzerland, 760–700). The antibody clone, catalogue number, vendor, dilution factor, antigen retrieval condition, antibody incubation time, and staining platform are summarized in Table S1. Briefly, the tissue sections were baked and deparaffinized, followed by epitope retrieval, then hydrogen peroxide blocking, then incubated with primary antibody and detection system. The antibody–antigen binding was visualized by brown DAB Refine stain and counter-stained blue with hematoxylin for 8 min. Tissue slides were then dehydrated offline, cleared in xylene, and cover-slipped using Cytoseal™ XYL solution. The stained slides were scanned with Leica Aperio AT2 slide scanner (Leica Biosystems, Deer Park, IL, USA) for analysis.

### 2.4. Immunohistochemistry (IHC) Scoring Methodology and Analysis

Anti-TF-1 antibody staining was performed by immunohistochemistry and evaluated in the membrane and cytoplasm of malignant cells and reported as histologic score (H-score) considering the extension (percentage) of positive expression at each intensity level (0, no staining; 1+, mild staining; 2+, moderate staining; 3+, strong staining) (range 0–300). H-scoring was also evaluated for nectin-4 and TROP2 membranous and cytoplasmic expression in malignant cells. The slides were primarily read by study pathologist (L.C.C.) with training in biomarker analysis in translational research who was blinded to clinical data, including HPV status. A second pathologist (L.S.S.) with expertise in molecular pathology and translational research reviewed a subset of samples included in the analysis and overall agreed with the scoring system. H-scores from all three cores were aggregated into a mean H-score. Any tissue with an H-score of 10 or higher was considered positive for TF and TROP2 [18,19], while H-score of 15 or higher was considered positive for nectin-4 [20,21]. Tumor protein p53 immunohistochemistry interpretation was performed based on the criteria initially outlined for vulvar cancer [22,23] and extended to penile cancer [24]. Normal p53 expression was characterized by either patchy nuclear staining with variable intensity or moderate to strong nuclear staining sparing the basal layer. Abnormal p53 expression was defined by one of four distinct patterns: (1) continuous strong and nuclear staining restricted to the basal layer, (2) diffuse strong nuclear staining in at least 80% of cells, (3) complete absence of nuclear staining in all tumor cells, with evidence of positive internal control, or (4) moderate to strong cytoplasmic staining. The p16 immunohistochemistry was evaluated based on the hybrid system (HS) previously described by Chahoud et al. [16]. Tumor sections were considered positive if one of the following staining patterns was present in more than 75% of neoplastic cells: (1) extensive discontinuous or (2) entire and continuous cytoplasmic or nuclear. Percentage of tumors staining positive for TF, TROP2, and nectin-4 and median H-scores were described and compared based on specimen HPV status, p53, and p16 staining. Table S2 summarizes IHC scoring of samples.

### 2.5. Study Endpoints and Follow-Up

Patients were followed from the time of definitive surgical intervention until the last recorded assessment in the medical record or death. Patients were grouped into one of 4 categories at the time of last follow-up: “Alive without disease”, “Alive with disease”, “Dead of disease”, and “Deceased.” Tumor specimen grade was classified using the American Joint Committee on Cancer (AJCC) 8th edition staging system criteria. Association of TF, TROP2, and nectin-4 expression with tumor grade, presence of metastatic disease, p53 staining pattern, p16 expression, lymphovascular invasion (LVI), perineural invasion (PNI), recurrence-free survival (RFS), and cancer-specific survival (CSS) was assessed. RFS was calculated as time from the date of surgery to the first recurrence (either local, regional, or distant) or death or censored at the last follow-up. CSS was calculated as time from the date of diagnosis to cancer-related death or censored at the last follow-up.

### 2.6. Statistical Analysis

Numerical variables are reported using the median, minimum, and maximum values. Categorical variables are reported with frequencies and percentages. Fisher’s exact test was used to assess association between categorical variables and TF, TROP2, and nectin-4 average staining scores, while the Wilcoxon rank sum test was used to compare numerical variables, which were expressed with median and interquartile range (IQR). Results for all associations determined using categorical variables are reported in Table S3. Spearman’s method was calculated to assess the degree of correlation (denoted as r) between the membrane and cytoplasm-specific average staining scores, respectively. Cox proportional hazard models were constructed to assess the association between the different H-scores, HPV, p16, and aberrant p53 status with RFS and CSS. Kaplan–Meier curves for RFS and CSS by HPV, p16, and aberrant p53 status were generated, along with Kaplan–Meier curves for RFS and CSS by TF expression stratified into the top (high) and bottom (low) 50th percentile H-scores. Due to the small sample size and exploratory nature of this study, no multivariable modeling was performed. All adjusted *p*-values were determined using Benjamini–Hochberg correction. For all tests, an α = 0.05 is used as the threshold for statistical significance. All computations were carried out using R version 4.4.1.

## 3. Results

### 3.1. Patient Baseline Characteristics

This study included 33 patients who underwent primary surgical treatment of PSCC at MDACC. Patient demographics for our cohort are provided in Table S4. The majority of these patients were Caucasian (60.6%) and underwent partial penectomy as their primary treatment (66.7%). Median age was 64 years (IQR 51.5–70). Pertinent patient history included significant tobacco use (60.6%), phimosis (57.6%), and lack of neonatal circumcision (12.1%). There was general concordance on tumor assessment, as most lesions were categorized as clinical stage T2 (51.5%) and N0 (57.6%) versus pathologic stage T2 (69.7%) and N0 (36.4%) on pre- and post-surgical analysis, respectively. Lymphovascular and perineural invasion were noted in 24 (72.7%) and 12 (36.4%) patients, respectively. Seventeen patients (51.5%) had HPV-positive disease, while 19 patients (57.6%) had metastatic disease. Patients were followed for a median of 19.3 months (IQR 5.5–42.2 months). Most patients were classified as “alive without disease” (42.4%) at the time of this study.

### 3.2. Study Endpoint Results

**Figure 1:**
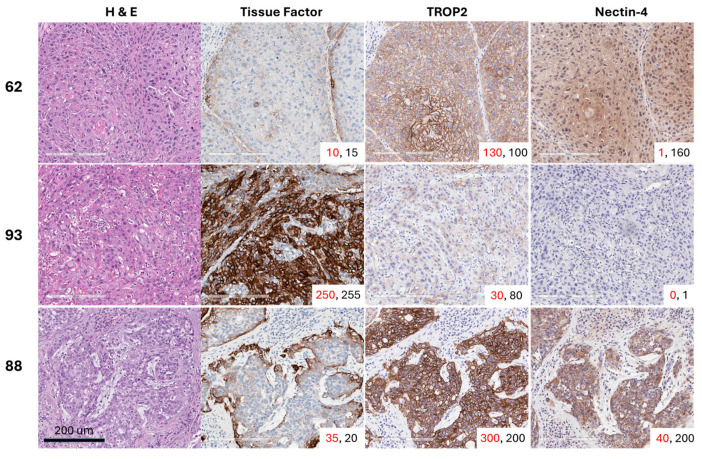
Representative images of three usual squamous cell carcinoma subtype PSCC tumor tissues with variable expression of tissue factor (TF), TROP2, and nectin-4. The membrane H-score is shown in red font, while the cytoplasmic H-score is displayed in black font for each core in the lower-right corner of each image.

Positive staining for TF was detected in 26 of 32 evaluable tumors (81.3%). Of the tumors that had H-scores < 10, five out of the 6 were HPV-positive (83.3%), and all 6 tissues were p16 positive. The median overall membrane TF H-score was 47 (range 1–232), while the median cytoplasmic H-score was slightly lower at 29 (range 2–180). All 32 tumors stained positive for TROP2. The TROP2 median membrane H-score was 106 (range 0–277), and the median cytoplasmic H-score was 109 (range 40–227). Twenty-six of the 32 tumors (81.3%) had cytoplasmic or membrane nectin-4 expression with an H-score ≥ 15. Only 15 of the 32 tumors (46.9%) were positive for nectin-4 by membrane staining, and the median H-score was 5.4 (range 0–137). Twenty-four of the 32 tumors (75.0%) were positive for nectin-4 by cytoplasmic staining, and the median H-score was 74 (range 0–172). Representative images of 3 usual squamous cell subtype PSCC tumor tissues from patients with grade 2 or 3 pT2 disease with variable expression in TF, TROP2, and nectin-4 are shown in Figure 1.

### 3.3. Associations with TF Expression

A significant association was observed between HPV status and TF expression. Staining was more prominent in HPV-negative tumors in both the membrane (median H-score 69.6 vs. 18.8; *p* = 0.003) and cytoplasm (median H-score 59.2 vs. 17.7, *p* = 0.007). A similar result was observed when comparing TF expression based on p16 status. Cytoplasmic (61.7 vs. 11.7, *p* < 0.001) and membrane TF staining (71.7 vs. 15.0, *p* < 0.001) favored p16-negative tumors. Final p53 status was more likely to be aberrant in the higher staining samples (cytoplasm: 61.7 vs. 18.3, *p* = 0.012; membrane: 67.5 vs. 20.3, *p* = 0.006). There was no association with either membrane [HR 1.0 (95% CI 0.99–1.01), *p* = 0.896] or cytoplasmic TF staining [HR 1 (95% CI 0.99–1.01), *p* = 0.729] when assessing CSS. Similarly, no association was observed when using RFS as an endpoint in both membrane [HR 1 (95% CI 0.99–1.01), *p* = 0.535] or cytoplasmic TF staining [HR 1 (95% CI 0.99–1.01), *p* = 0.514]. There were no significant associations linking TF H-score to PNI, LVI, metastasis, or primary tumor grade.

### 3.4. Associations with TROP2/Nectin-4 Expression

Unlike TF, we did not observe any association with TROP2 and nectin-4 staining with aberrant p53 or HPV status. We observed an association of TROP2 staining with positive LVI (membrane median H-score 136.7 vs. 66.7, *p* = 0.014; cytoplasmic median H-score 110 vs. 93.3, *p* = 0.04). We observed an association with TROP2 staining and positive p16 status (membrane 120.3 vs. 85, *p* = 0.052; cytoplasmic 135 vs. 107.5, *p* = 0.041). Similar to TF, we did not observe any correlations between RFS and CSS with TROP2 or nectin-4 staining.

### 3.5. Correlations Between Surface Proteins and Other Factors

**Figure 2:**
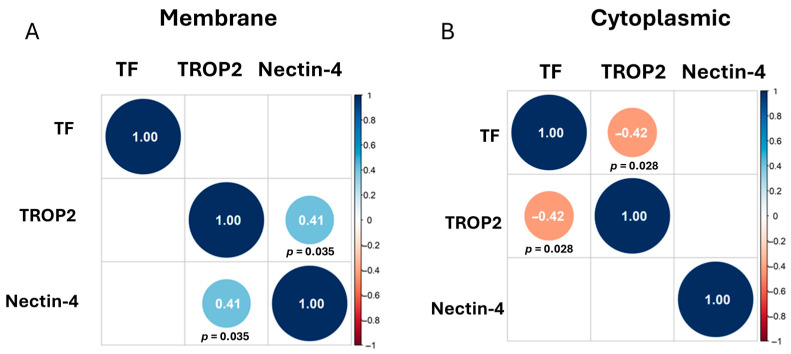
Correlation Matrices. Correlations that are off the diagonal are statistically significant, with positive correlations shown in light blue circles and negative correlations shown in orange circles. Adjusted *p*-values were determined using Benjamini–Hochberg correction and are written below diagonal circles. Correlation plot of the membrane staining (**A**) and of the cytoplasmic staining (**B**) of TF, TROP2, and nectin-4 shown.

We estimated the Spearman correlation between TF, nectin-4, and TROP2 using the membrane staining scores (Figure 2A) and cytoplasmic staining scores (Figure 2B). Correlations that are off the diagonal are statistically significant. We observed a positive correlation between TROP2 and nectin-4 membrane staining (r = 0.41), while we observed a negative correlation between cytoplasmic TF and TROP2 staining (r = −0.42).

Contingency tables were utilized to determine the association between aberrant p53 and HPV status, aberrant p53 and p16 status, and p16 and HPV status (Table 1). The *p*-values obtained from the Fisher’s exact test confirmed that the three sets of variables were all associated with each other. In addition, we determined the association of p16, HPV, and aberrant p53 status with CSS and RFS. Table 2 shows these associations along with hazard ratios (HR). Positive HPV and p16 status are marginally associated with RFS at an α = 0.103, both exhibiting a protective effect on recurrence. Aberrant p53 is associated with RFS (adjusted *p*-value = 0.020) and has a negative effect on RFS. For CSS, positive HPV and p16 status are marginally associated. As with RFS, positive HPV and p16 status exhibit a protective effect for CSS. For p53 and CSS, the large HR coefficient is probably due to the low number of cancer-related deaths with normal p53 staining (only one death). Kaplan–Meier curves for RFS and CSS by HPV, p16, and aberrant p53 status were generated, along with Kaplan–Meier curves for RFS and CSS by TF expression stratified into the top (high) and bottom (low) 50th percentile H-scores (Figures S1 and S2).

## 4. Discussion

This study examined a cohort of advanced PSCC patients with well-documented clinical outcomes to assess TF expression in primary tumor specimens. We found significant TF positivity in 81.3% of the cases using a cutoff of ≥10% of tumor cells with at least 1+ intensity in cytoplasm and/or membrane. We found TF expression was highly associated with p16 and HPV-negative tumors and aberrant p53 staining. Similar results were not observed with TROP2 and nectin-4 staining. In addition, a negative correlation was observed with membrane TF and TROP2 staining, indicating that these two surface proteins could be expressed in two different subsets of patients with TROP2 in HPV-positive tissues and TF in HPV-negative tissues. Our results indicate that TF expression could be a positive biomarker for HPV-independent advanced PSCC, which is currently determined by the lack of p16 staining and a negative test for HPV using either PCR or in situ hybridization assays. HPV-independent PSCC has been shown to be associated with *TP53* mutations [24,25,26,27]. The results from contingency Table 1 validate this assumption, as we see aberrant p53 is associated with negative p16 and HPV status. Our TF data suggests that TF could be either a driver or passenger in *TP53*-mutated HPV-independent advanced PSCC.

TF expression has been found to be regulated by multiple transcription factors and signaling pathways [28,29]. In colorectal cancer (CRC), isogenic cell lines were shown to express higher TF with mutant K-ras and loss-of-function mutations in p53 [29]. The same study found TF expression to be an important driver in CRC progression by observing increased tumor growth in TF-high CRC, slower growth with TF siRNA-expressing CRC clones, and higher expression of anti-angiogenic markers TSP-1 and TSP-2 in TF-silenced tumors in mouse models. This relationship between p53 and TF expression was observed in CRC patient tumor samples. By IHC, TF expression was found in all tissues with a loss of function genetic *TP53* mutation (48/48) and to have higher staining than tissues with wildtype *TP53* [30]. In non-small cell lung cancer, TF gene expression was associated with reduced survival and was higher in samples with mutated *TP53* [31,32]. The mechanistic relationship between mutant p53 and TF expression represents a key gap in current knowledge in advanced PSCC.

Our TF values are on par with the reported rates of TF expression in other solid tumors highlighted in a recent study by de Bono et al. [18]. Criteria for positive TF expression in that study were similar to our study and were characterized by modified H-scores. It should be noted that a number of tumors analyzed in the study by de Bono et al. utilized tissue obtained from biopsy as opposed to completely excised tissue which may have impacted the observed positivity rates. Interestingly, cervical cancer, which has similar pathogenesis to PSCC, showed TF expression of 77%. Other studies have also corroborated this increased expression in cervical cancer and described an association of increased expression with worse outcomes [33]. It is important to note that unlike PSCC, which is only associated with high-risk HPV infection in up to 50% of cases, cervical cancer is almost universally associated with infection with high-risk HPV [34].

To our knowledge, this is the first study to date highlighting TF expression in advanced penile cancer and further increased TF expression in HPV-negative, aberrant p53 advanced PSCC specifically. The majority of studies exploring novel targets in PSCC have largely focused on HPV-positive PSCC. Grass et al. first described elevated expression of nectin-4, an immunoglobulin involved in cell–cell junction formation and maintenance, in invasive PSCC in 2022 [11]. Programmed death ligand-1 (PD-L1) and TROP2 have also been examined, with associations found between these proteins and higher tumor stage and disease progression, respectively [10]. Within this study, increased expression of both TROP2 and nectin-4 was found in HPV-positive cases, which was not observed in our study. This could possibly stem from our use of a TMA, while the other study used whole slides. Heterogeneity of staining within tumor tissues of surface proteins TROP2 and nectin-4 was noted in [10] and in other IHC studies [35]. In addition, the discrepancy could also be related to our lower sample size (121 versus 33). In our study, we did not find a significant association of TF H-score with tumor grade, presence of metastatic disease, LVI, PNI, CSS, or RFS. Previous literature has detailed the association of TF expression with poor prognosis in other solid tumors [36]. Associations between TF overexpression and metastases in breast cancer, non-small cell lung cancer, and colorectal cancer have been reported [37,38,39]. Furthermore, increased TF expression in colorectal cancer has specifically been linked to the development of hepatic metastasis, which carries a particularly grim prognosis [40]. TF was not associated with RFS, CSS, or metastatic progression in our study; however, this may be related to the small sample size. The limitations of a small sample size in our study are evident in the large, calculated HR for aberrant p53 status in CSS, as it is driven by the fact that only one cancer-related death occurred in the p53-negative cohort. However, even in this small cohort, aberrant p53 was associated with worse RFS and CSS. Our data is consistent with a recent report from Elst et al. revealing that aberrant p53 tumor expression was associated with worse CSS among a cohort of 541 subjects irrespective of HPV status [41]. Given that TF identifies the aberrant p53 cohort and represents a viable therapeutic target, the current study is particularly relevant and warrants further investigation of TF expression and its association with PSCC progression in a larger patient cohort.

As previously stated, prognosis in advanced PSCC remains poor in those whose disease has progressed on frontline platinum-based systemic therapy. These outcomes are decidedly worse in those who present with HPV-independent disease. Therapeutic targets for HPV-negative PSCC are sparse in the literature. The novel finding of increased TF expression in this population may present an ideal therapeutic target, as treatment in other squamous tumors has yielded notable results. For example, cervical cancer has also been demonstrated to have TF expression of up to 77%, which exceeds expression in other solid tumors [18]. Tisotumab vedotin (TV) is a TF-specific antibody–drug conjugate paired with monomethyl auristatin E (MMAE) that works to inhibit microtubule activity, thus enacting a direct cytotoxic effect [42]. Coleman et al. conducted a multicenter phase II trial utilizing TV in patients with recurrent or metastatic cervical cancer who had progressed on or after doublet chemotherapy with bevacizumab and had two or fewer previous systemic regimens for their disease in the frontline setting. This study showed a confirmed objective response rate of 24%, with 28% of study patients reporting grade 3 or worse treatment-related adverse events (TRAEs) [14]. A follow-up phase 3 study examining TV as second- or third-line therapy in patients with recurrent or metastatic cervical cancer demonstrated a similar improvement in confirmed objective response rate and TRAEs, in addition to a 30% lower risk of death and improved progression-free survival in the TV cohort when compared with standard chemotherapy regimens [15]. These results support the safety of TV and other ADCs in the clinical setting and their potential for use in advanced PSCC in the second-line setting and beyond. In locally advanced cases, patients who show a significant response may also be more likely to benefit from consolidative therapies (e.g., radical surgical resection or radiation).

There are several limitations of this study. Firstly, the sample size of only 33 patients is relatively small but consistent with the rarity of PSCC. Secondly, the most recent primary surgery from the study occurred in 2016, which makes all samples 8+ years old at the time of the analysis. This may introduce time-based degradation of the antigens in question and may have impacted the fidelity of immunohistochemical staining and scoring. Lastly, utilizing TMAs in our analysis may not have fully accounted for tumor heterogeneity, as this represents a small sample of the overall malignancy, in contrast to examination of whole tissue slides.

Notwithstanding the above limitations, we have clearly shown that TF is expressed in the majority of advanced PSCC specimens in our cohort. This demonstration of TF expression in advanced PSCC that is enhanced in the HPV-negative p53-aberrant cohort is a promising discovery that could have important treatment implications in the future. Preclinical studies will be of interest to further delineate the benefit of targeting TF in advanced PSCC with eventual transition to clinical trials in this space. This is already being explored with other ADCs in PSCC with great anticipation (NCT06104618).

## 5. Conclusions

TF expression was noted among 81.3% of advanced PSCC tissues in our study. Expression was significantly higher among the HPV- and p16-negative cohort and the aberrant p53 cohort. These data support further investigation of TF-targeted therapy, such as an ADC in advanced PSCC, especially among the HPV-negative p53 aberrant cohort.

## Figures and Tables

**Table 1:** Contingency tables illustrating the associations between aberrant p53 and HPV status, aberrant p53 and p16 status, and p16 and HPV status. Adjusted *p*-values were determined using Benjamini–Hochberg correction.

**Patient Characteristics**	**Total**	**HPV** **Negative**	**HPV** **Positive**	**Adjusted *p*-Value**
p53 status normal aberrant	21 (65.6%) 11 (34.4%)	7 (43.8%) 9 (56.2%)	14 (87.5) 2 (12.5%)	0.023
p16 status negative positive	17 (53.1%) 15 (46.9%)	15 (93.8%) 1 (6.3%)	2 (12.5%) 14 (87.5%)	< 0.001
**Patient characteristics**	**Total**	**p16** **negative**	**p16** **positive**	**Adjusted *p*-value**
p53 status normal aberrant	21 (65.6%) 11 (34.4%)	6 (35.3%) 11 (64.7%)	15 (100) 0 (0%)	< 0.001

**Table 2:** Associations with RFS and CSS with HR for HPV, p16, and aberrant p53 status. Adjusted *p*-values were determined using Benjamini–Hochberg correction. * Reference for each association is the second category listed. ** The large HR is from the small number (1 death) in the normal p53 group.

**Recurrence-Free Survival (RFS)**			
**Variable**	Level *	**HR (95% Cl for HR)**	**Adjusted *p*-Value**
HPV	positive vs. negative	0.45 (0.17–1.17)	0.103
p16	positive vs. negative	0.4 (0.15–1.07)	0.103
p53	aberrant vs. normal	4.06 (1.48–11.15)	0.020
**Cancer-Specific Survival (CSS)**			
**Variable**	**Level**	**HR (95% Cl for HR)**	**Adjusted *p*-value**
HPV	positive vs. negative	0.23 (0.04–1.2)	0.121
p16	positive vs. negative	0.09 (0.01–0.73)	0.073
p53	aberrant vs. normal	4683379200.32 (0-Inf) **	1.00

## Data Availability

The data presented in this study are available upon reasonable request from the corresponding author.

## Supplementary Materials

The following supporting information can be downloaded at: https://www.mdpi.com/article/10.3390/cancers17213410/s1. Table S1. The antibody clone, catalogue number, vendor, dilution factor, antigen retrieval condition, antibody incubation time, and staining platform are summarized. Table S2. Summary of H-score criteria and cutoffs used to define staining status of tissue factor (TF), nectin-4, TROP2, p53, and p16 along with the number of evaluable tumors for each marker and the reference used to define the criteria. Table S3. Associations determined using average H-score of TF, TROP2, and nectin-4 with PNI, LVI, p53, HPV, primary tumor grade, and p16. Table S4. Descriptive statistics of patient population. The percentage is given in the parentheses. Figure S1. Recurrence-Free Survival (RFS) Kaplan–Meier curves for (A) HPV status, (B) p16 status, and (C) aberrant p53 status. Kaplan–Meier curve by TF expression stratified into the top (high) and bottom (low) 50th percentile H-scores for both membrane (D) and cytoplasmic (E) staining. Figure S2. Cancer-Specific Survival (CSS) Kaplan–Meier curves for (A) HPV status, (B) p16 status, and (C) aberrant p53 status. Kaplan–Meier curve by TF expression stratified into the top (high) and bottom (low) 50th percentile H-scores for both membrane (D) and cytoplasmic (E) staining.

## Abbreviations

The following abbreviations are used in this manuscript:

ADC	Antibody-drug conjugate
AJCC	American Joint Committee on Cancer
CSS	Cancer-specific survival
FFPE	Formalin-fixed paraffin-embedded
H&E	Hematoxylin and eosin
HPV	Human papillomavirus
JAK	Janus kinase
LVI	Lymphovascular invasion
MDACC	MD Anderson Cancer Center
MMAE	Monomethyl auristatin E
PNI	Perineural invasion
PSCC	Penile squamous cell carcinoma
RFS	Recurrence-free survival
STAT	Signal transducer and activator of transcription
TF	Tissue factor
TIP	Paclitaxel, ifosfamide, and cisplatin
TMA	Tissue microarray
TRAE	Treatment-related adverse event
TROP2	Trophoblast cell-surface antigen 2
TV	Tisotumab vedotin
