# Supplementary material for: Tissue Factor Expression in Penile Squamous Cell Carcinoma: A Potential Marker of HPV-Independent Disease
Source: Cancers (Basel). 2025 Oct 23;17(21):3410. doi: 10.3390/cancers17213410 (PMC12606763; doi:10.3390/cancers17213410)
Supplement: Supplementary file 1 [file cancers-17-03410-s001.zip › cancers-3825177-supplementary.pdf]

Antibody	Clone	Vendor	Cat#	Dilution Factor	Antigen Retrieval	Antibody Incubation Time	AutoStainer
**TROP2**	EPR20043m	Abcam	ab214486	1:4000	ER1 for 20 minutes at 100°C	15 minutes	Leica Bond III platform
**NECTIN-4**	EPR15613-68	Abcam	ab192033	1:1000	ER2 for 10 minutes at 100°C	15 minutes	Leica Bond III platform
**P16**	E6H4	Ventana	705-4793	Ready to Use	CC1 for 48 minutes at 100°C	32 minutes	Benchmark Ultra Platform
**P53**	DO-7	Leica	PA0057	1:2	ER2 for 20 minutes at 100°C	15 minutes	Leica Bond III platform
**CD142**	HTF-1	Thermo Fisher	#16-1429-82	1:200	CC1 for 80 minutes at 100°C	32 minutes	Discovery Ultra Platform

Note: ER1: Epitope Retrieval solution 1, pH 6.0; ER2: Epitope Retrieval solution 2, pH9.0; CC1: Cell condition Ultra, pH8.0.

Table S1. The antibody clone, catalogue number, vendor, dilution factor, antigen retrieval condition, antibody incubation time, and staining platform are summarized.

Marker	Scoring Criteria	N	Reference
Tissue factor (TF)	H-score determined by considering the extension (percentage) of positive expression at each intensity level (0, no staining, 1+ mild staining, 2+ moderate staining, 3+ strong staining) (range 0-300) of each core. Tissue considered TF positive if aggregated mean H-score was ≥ 10, membranous or cytoplasmic.	32	[1]
Nectin-4	H-score determined by considering the extension (percentage) of positive expression at each intensity level (0, no staining, 1+ mild staining, 2+ moderate staining, 3+ strong staining) (range 0-300) of each core. Tissue considered nectin-4 positive if aggregated mean H-score was ≥ 15, membranous or cytoplasmic.	32	[2,3]
TROP2	H-score determined by considering the extension (percentage) of positive expression at each intensity level (0, no staining, 1+ mild staining, 2+ moderate staining, 3+ strong staining) (range 0-300) of each core. Tissue considered TROP2 positive if aggregated mean H-score was ≥ 10, membranous or cytoplasmic.	32	[4]
p53	Abnormal p53 expression was defined by one of four distinct patterns: (1) continuous strong and nuclear staining restricted to the basal layer, (2) diffuse strong nuclear staining least of at least 80% of cells, (3) complete absence of nuclear staining in all tumor cells, with evidence of positive internal control, or (4) moderate to strong cytoplasmic staining.	32	[5,6]
p16	Positive if one of the following staining patterns was present in more than 75% of neoplastic cells (1) extensive discontinuous or (2) entire and continuous cytoplasmic or nuclear staining.	32	[7]

Table S2. Summary of H-score criteria and cutoffs used to define staining status of tissue factor (TF), nectin-4, TROP2, p53, and p16 along with the number of evaluable tumors for each marker and the reference used to define the criteria.

PNI

Patient Characteristics		Total N=32	no N=10	yes N=12	P-value
Membrane Tissue Factor Average Final H Score, median (min,max)	N = 32	46.92 (0.67, 231.67)	69.59 (5.67, 136.67)	43.75 (4, 231.67)	0.722
Cytoplasm Tissue Factor Average Final H Score, median (min,max)	N = 32	29.16 (2, 180)	33.5 (5, 98.33)	31.09 (3, 180)	0.921
Trop 2 Membrane Average Final H Score, median (min,max)	N = 32	106.66 (0, 276.67)	111.66 (66.67, 276.67)	98.34 (0, 276.67)	0.276
Trop 2 Cytoplasm Average Final H Score, median (min,max)	N = 32	109.16 (40, 226.67)	111.66 (93.33, 226.67)	100 (40, 210)	0.106
Nectin 4 Membrane Average Final H Score, median (min,max)	N = 32	5.42 (0, 136.67)	10.16 (0, 69.33)	3.33 (0, 115)	0.712
Nectin 4 Cytoplasm Average Final H Score, median (min,max)	N = 32	74.16 (0, 171.67)	90 (3.33, 166.67)	58.34 (0, 168.33)	0.337

LVI

Patient Characteristics		Total N=32	no N=7	yes N=23	P-value
Membrane Tissue Factor Average Final H Score, median (min,max)	N = 32	46.92 (0.67, 231.67)	47.5 (17.5, 231.67)	46.33 (1.33, 205)	0.471
Cytoplasm Tissue Factor Average Final H Score, median (min,max)	N = 32	29.16 (2, 180)	30.5 (18.33, 180)	28.33 (2.33, 123.33)	0.202
Trop 2 Membrane Average Final H Score, median (min,max)	N = 32	106.66 (0, 276.67)	66.67 (0, 105)	136.67 (15, 276.67)	0.014
Trop 2 Cytoplasm Average Final H Score, median (min,max)	N = 32	109.16 (40, 226.67)	93.33 (40, 136.67)	110 (73, 226.67)	0.041
Nectin 4 Membrane Average Final H Score, median (min,max)	N = 32	5.42 (0, 136.67)	0 (0, 69.33)	17 (0, 136.67)	0.117
Nectin 4 Cytoplasm Average Final H Score, median (min,max)	N = 32	74.16 (0, 171.67)	46.67 (0, 90)	90 (0, 171.67)	0.073

p53 Status

Patient Characteristics		Total N=32	normal N=21	aberrant N=11	P-value
Membrane Tissue Factor Average Final H Score, median (min,max)	N = 32	46.92 (0.67, 231.67)	20.33 (0.67, 205)	67.5 (23.67, 231.67)	0.006
Cytoplasm Tissue Factor Average Final H Score, median (min,max)	N = 32	29.16 (2, 180)	18.33 (2, 123.33)	61.67 (25, 180)	0.012
Trop 2 Membrane Average Final H Score, median (min,max)	N = 32	106.66 (0, 276.67)	120.33 (15, 276.67)	85 (0, 225)	0.077
Trop 2 Cytoplasm Average Final H Score, median (min,max)	N = 32	109.16 (40, 226.67)	110 (73, 226.67)	107.5 (40, 145)	0.274
Nectin 4 Membrane Average Final H Score, median (min,max)	N = 32	5.42 (0, 136.67)	3.33 (0, 136.67)	7.5 (0, 115)	0.456
Nectin 4 Cytoplasm Average Final H Score, median (min,max)	N = 32	74.16 (0, 171.67)	88.33 (0, 171.67)	46.67 (0, 126.67)	0.131

HPV status

Patient Characteristics		Total N=32	negative N=16	positive N=16	P-value
Membrane Tissue Factor Average Final H Score, median (min,max)	N = 32	46.92 (0.67, 231.67)	69.59 (1.33, 231.67)	18.75 (0.67, 123.33)	0.003
Cytoplasm Tissue Factor Average Final H Score, median (min,max)	N = 32	29.16 (2, 180)	59.17 (2.33, 180)	17.66 (2, 98.33)	0.007
Trop 2 Membrane Average Final H Score, median (min,max)	N = 32	106.66 (0, 276.67)	96.66 (0, 225)	107.5 (15, 276.67)	0.207
Trop 2 Cytoplasm Average Final H Score, median (min,max)	N = 32	109.16 (40, 226.67)	107.91 (40, 145)	124.16 (85.33, 226.67)	0.073
Nectin 4 Membrane Average Final H Score, median (min,max)	N = 32	5.42 (0, 136.67)	12.09 (0, 136.67)	3.33 (0, 80)	0.618
Nectin 4 Cytoplasm Average Final H Score, median (min,max)	N = 32	74.16 (0, 171.67)	85.83 (0, 171.67)	65 (0, 168.33)	0.720

Primary Tumor Grade

Patient Characteristics		Total N=32	2 N=13	3 N=19	P-value
Membrane Tissue Factor Average Final H Score, median (min,max)	N = 32	46.92 (0.67, 231.67)	47.5 (4, 205)	46.33 (0.67, 231.67)	0.323
Cytoplasm Tissue Factor Average Final H Score, median (min,max)	N = 32	29.16 (2, 180)	28.33 (3, 123.33)	30 (2, 180)	0.367
Trop 2 Membrane Average Final H Score, median (min,max)	N = 32	106.66 (0, 276.67)	93.33 (0, 185)	136.67 (15, 276.67)	0.053
Trop 2 Cytoplasm Average Final H Score, median (min,max)	N = 32	109.16 (40, 226.67)	106.67 (90, 145)	110 (40, 226.67)	0.513
Nectin 4 Membrane Average Final H Score, median (min,max)	N = 32	5.42 (0, 136.67)	15 (0, 69.33)	3.33 (0, 136.67)	>0.99
Nectin 4 Cytoplasm Average Final H Score, median (min,max)	N = 32	74.16 (0, 171.67)	60 (0, 125)	83.33 (0, 171.67)	0.233

p16 Status

Patient Characteristics		Total N=32	negative N=17	positive N=15	P-value
Membrane Tissue Factor Average Final H Score, median (min,max)	N = 32	46.92 (0.67, 231.67)	71.67 (23.67, 231.67)	15 (0.67, 108.33)	<0.001
Cytoplasm Tissue Factor Average Final H Score, median (min,max)	N = 32	29.16 (2, 180)	61.67 (15.67, 180)	11.67 (2, 98.33)	<0.001
Trop 2 Membrane Average Final H Score, median (min,max)	N = 32	106.66 (0, 276.67)	85 (0, 225)	120.33 (15, 276.67)	0.052
Trop 2 Cytoplasm Average Final H Score, median (min,max)	N = 32	109.16 (40, 226.67)	107.5 (40, 145)	135 (85.33, 226.67)	0.041
Nectin 4 Membrane Average Final H Score, median (min,max)	N = 32	5.42 (0, 136.67)	7.5 (0, 115)	3.33 (0, 136.67)	0.939
Nectin 4 Cytoplasm Average Final H Score, median (min,max)	N = 32	74.16 (0, 171.67)	70 (0, 126.67)	78.33 (0, 171.67)	0.596

Table S3. Associations determined using average H-score of TF, TROP2, and nectin-4 with PNI, LVI, p53, HPV, primary tumor grade, and p16.

	Total (n=33)	HPV Positive (n=17)	HPV Negative (n=16)	p-value
Race/Ethnicity (%)				0.31
Caucasian/White	20 (60.6)	11 (64.7)	9 (56.3)	
African American/Black	3 (9.1)	3 (17.7)		
Hispanic	10 (30.3)	3 (17.7)	7 (43.8)	
Median Age (years, IQR)	64 (51.5 – 70)	66 (54 – 69.5)	58 (38 – 70)	0.49
Median BMI (IQR)	30.7 (22.5 – 48)	29.4 (28.2 – 38.3)	32 (26.8 – 37)	0.98
Median Follow-up (months; IQR)	19.3 (5.5 – 42.2)	21.4 (7.2 – 37.3)	18.0 (5.4 – 56.1)	0.94
P53				0.66
Yes	25 (75.8)	14 (82.4)	11 (68.8)	
No	8 (18.4)	3 (17.7)	5 (31.3)	
LVI (%)				0.96
Yes	24 (72.7%)	12 (70.6)	12 (75.0)	
No	9 (27.3)	5 (29.4)	4 (25.0)	
PNI (%)				0.92
Yes	12 (36.4)	5 (29.4)	7 (43.8)	
No	10 (30.3)	5 (29.4)	5 (31.3)	
N/a	11 (33.3)	7 (41.2)	4 (25.0)	
Metastasis (%)				0.42
Yes	19 (57.6)	7 (41.2)	10 (58.8)	
No	14 (42.4)	10 (58.8)	6 (37.5)	
Tumor grade (%)				0.98
G2	13 (39.4)	7 (41.2)	6 (37.5)	
G3	20 (60.6)	10 (58.8)	10 (58.8)	
Clinical tumor stage (%)				0.66
T1	6 (18.2)	1 (5.9)	5 (31.3)	
T2	17 (51.5)	10 (58.8)	7 (43.8)	
T3	8 (24.2)	5 (29.4)	3 (18.8)	
T4	1 (3.0)		1 (6.3)	
Tx	1 (3.0)	1 (5.9)		
Clinical nodal stage				0.33
N0	19 (57.6)	11 (64.7)	8 (50.0)	
N1	9 (27.3)	2 (11.8)	7 (43.8)	
N2	3 (9.1)	3 (17.7)		
N3	1 (3.0)	1 (5.9)		
Nx	1 (3.0)		1 (6.3)	
Pathologic tumor stage				>0.99
T2	23 (69.7)	12 (70.6)	11 (68.8)	
T3	9 (27.3)	5 (29.4)	4 (25.0)	
T4	1 (3.0)		1 (6.3)	
Pathologic nodal stage				0.22
N0	12 (36.4)	10 (58.8)	2 (12.5)	
N1	7 (21.2)	3 (17.7)	3 (18.8)	
N2	4 (12.1)	1 (5.9)	2 (12.5)	
N3	8 (24.2)	2 (11.8)	8 (50.0)	
Nx	2 (6.1)	1 (5.9)	1 (6.3)	
Primary Procedure (%)				0.97
Partial penectomy	22 (66.7)	11 (64.7)	11 (68.8)	
Total penectomy	11 (33.3)	6 (35.3)	5 (31.3)	
Histology (%)				0.41
Usual	28 (84.9)	13 (76.5)	15 (93.8)	
Basaloid	5 (15.2)	4 (23.5)	1 (6.3)	
Recurrence (%)				0.01
Yes	9 (27.3)	4 (23.5)	11 (68.8)	

No	24 (72.7)	13 (76.5)	5 (31.3)	
Survival (%)				0.30
Alive	17 (51.5)	11 (64.7)	6 (37.5)	
Dead	16 (48.5)	6 (35.3)	10 (58.8)	

Table S4. Descriptive statistics of patient population. Percentage is given in the parentheses.

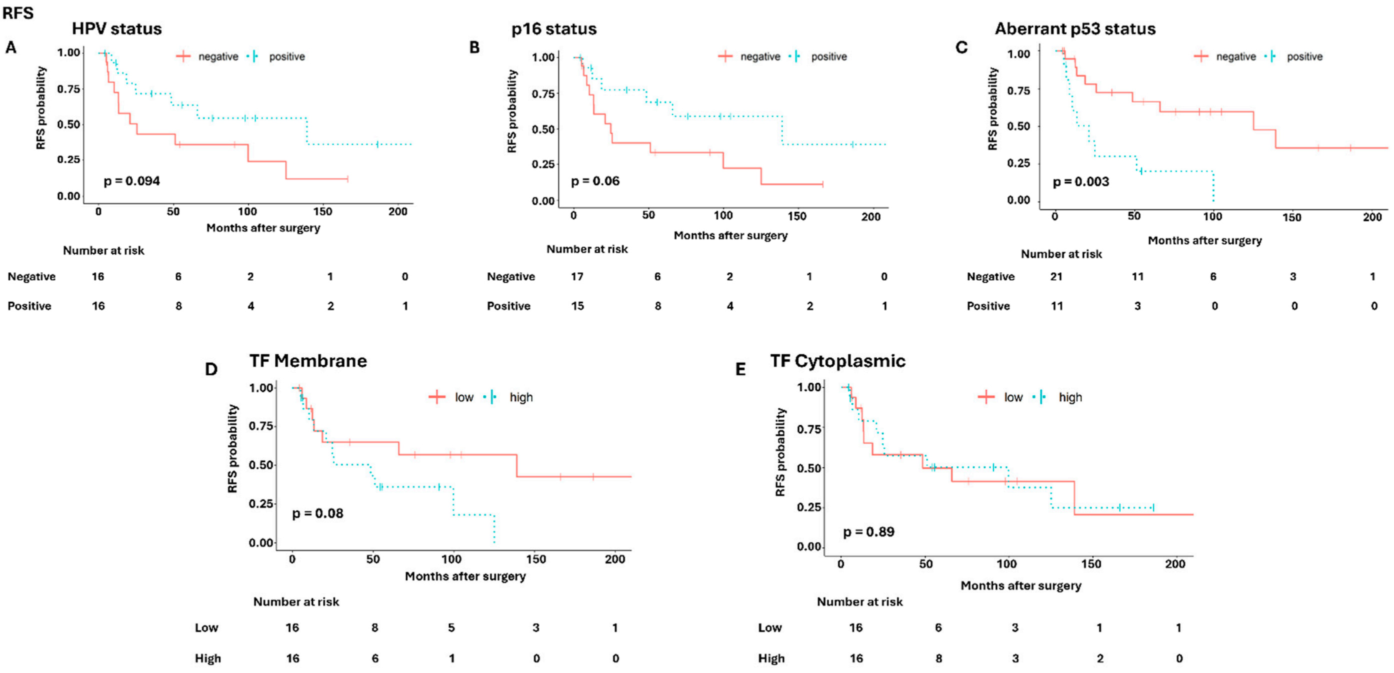

Figure S1. Recurrence Free Survival (RFS) Kaplan Meier curves for (A) HPV status, (B) p16 status, and (C) aberrant p53 status. Kaplan Meier curve by TF expression stratified into the top (high) and bottom (low) 50th percentile H-scores for both membrane (D) and cytoplasmic (E) staining.

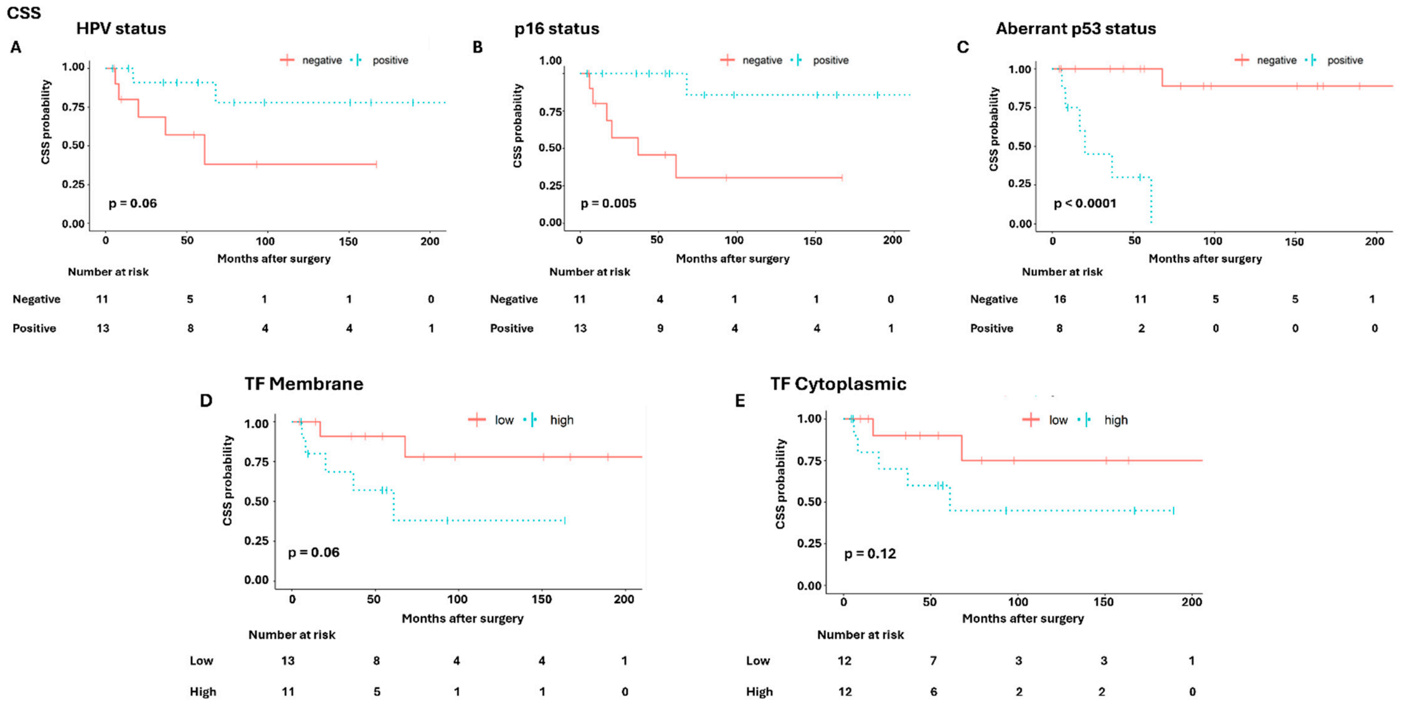

Figure S2. Cancer Specific Survival (CSS) Kaplan Meier curves for (A) HPV status, (B) p16 status, and (C) aberrant p53 status. Kaplan Meier curve by TF expression stratified into the top (high) and bottom (low) 50th percentile H-scores for both membrane (D) and cytoplasmic (E) staining.

References

1. de Bono J.S.; Harris J.R.; Burm S.M.; Vanderstichele A.; Houtkamp M.A.; Aarass S.; Riisnaes R.; Figueiredo I.; Nava Rodrigues D.; Christova R.; et al. Systematic study of tissue factor expression in solid tumors. *Cancer Rep (Hoboken)* **2023**, 6(2), e1699.
2. Hoffman-Censits J.H.; Lombardo K.A.; Parimi V.; Kamanda S.; Choi W.; Hahn N.M.; McConkey D.J.; McGuire B.M.; Bivalacqua T.J.; Kates M.; Matoso A. Expression of Nectin-4 in Bladder Urothelial Carcinoma, in Morphologic Variants, and Nonurothelial Histotypes. *Appl Immunohistochem Mol Morphol* **2021**, 29(8), 619-25.
3. Klumper N.; Tran N.K.; Zschabitz S.; Hahn O.; Buttner T.; Roghmann F.; Bolenz C.; Zengerling F.; Schwab C.; Nagy D.; et al. NECTIN4 Amplification Is Frequent in Solid Tumors and Predicts Enfortumab Vedotin Response in Metastatic Urothelial Cancer. *J Clin Oncol* **2024**, 42(20), 2446-55.
4. Jeon Y.; Jo U.; Hong J.; Gong G.; Lee H.J. Trophoblast cell-surface antigen 2 (TROP2) expression in triple-negative breast cancer. *BMC Cancer* **2022**, 22(1), 1014.
5. Tessier-Cloutier B.; Kortekaas K.E.; Thompson E.; Pors J.; Chen J.; Ho J.; Prentice L.M.; McConechy M.K.; Chow C.; Proctor L.; et al. Major p53 immunohistochemical patterns in in situ and invasive squamous cell carcinomas of the vulva and correlation with TP53 mutation status. *Mod Pathol* **2020**, 33(8), 1595-605.
6. Kobel M.; Kang E.Y. The Many Uses of p53 Immunohistochemistry in Gynecological Pathology: Proceedings of the ISGyP Companion Society Session at the 2020 USCAP Annual9 Meeting. *Int J Gynecol Pathol* **2021**, 40(1), 32-40.
7. Chahoud J.; Zacharias N.M.; Pham R.; Qiao W.; Guo M.; Lu X.; Alaniz A.; Segarra L.; Martinez-Ferrer M.; Gleber-Netto F.O.; et al. Prognostic Significance of p16 and Its Relationship with Human Papillomavirus Status in Patients with Penile Squamous Cell Carcinoma: Results of 5 Years Follow-Up. *Cancers (Basel)* **2022**, 14(24), 6024.
